# Activities of three erythrocyte enzymes of hyperglycemic rats (*Rattus norvegicus*) treated with *Allium sativa* extract

**DOI:** 10.1186/2251-6581-13-50

**Published:** 2014-04-22

**Authors:** Paul C Chikezie, Augustine A Uwakwe

**Affiliations:** 1 Department of Biochemistry, Imo state university, Owerri 460222, Nigeria; 2 Department of Biochemistry, University of Port Harcourt, Port Harcourt 460222, Nigeria

**Keywords:** Glutathione S-transferases, NADH-Methaemoglobin reductase, Na^+^/K^+^-ATPase, *Allium sativa*, Hyperglycemia, Diabetes mellitus

## Abstract

**Background:**

The present study sought to investigate erythrocyte glutathione S-transferases (GST), NADH-Methaemoglobin reductase (NADH-MR) and Na^+^/K^+^-ATPase activities of hypoglycemic rats treated with ethanol/water (1:2 *v/v*) extract of *A. sativa* as agent of glycemic control.

**Methods:**

Hyperglycemia was induced by a single intra-peritoneal injection of 0.1 mol/L alloxan monohydrate in phosphate buffer saline (PBS) solution (pH = 7.4); dosage = 140 mg/kg. At the end of the experimental time (*t* = 76 h), erythrocyte GST, NADH-MR and Na^+^/K^+^-ATPase activities as well as serum fasting blood sugar (FBS) levels were measured by spectrophotometric methods.

**Results:**

Serum FBS levels of control/normal (C/N) rats ranged between 72.93 ± 0.82–95.12 ± 0.92 mg/dL, whereas experimental rats without glycemic control gave: 249.41 ± 1.03–256.11 ± 1.23 mg/dL. Hyperglycemic rats treated with ethanol/water (1:2 *v/v*) extract of *A. sativa* exhibited comparative reduced serum levels of FBS alongside with erythrocyte GST, NADH-MR and Na^+^/K^+^-ATPase activities. The average relative activities of the three enzymes and corresponding order of enzyme activity in hyperglycemic rats treated with ethanol/water (1:2 *v/v*) extract of *A. sativa* was: NADH-MR = 60.99% > GST = 47.81% > Na^+^/K^+^-ATPase = 46.81%. In the same order, relative activities of the three enzymes in rats without glycemic control were: NADH-MR = 49.65% > GST = 23.69% > Na^+^/K^+^-ATPase = 17.02%.

**Conclusion:**

Erythrocyte GST, NADH-MR and Na^+^/K^+^-ATPase activities gave insights into the pathophysiology of diabetic state and served as biomarkers for ascertaining therapeutic control in Type 1 diabetes mellitus.

## Introduction

Diabetic mellitus is an endocrine disorder characterized by insufficiency in circulating plasma level of insulin (Type 1, or Insulin-Dependent Diabetes Mellitus; IDDM) and peripheral resistance and insensitivity to insulin (Type 2, or Non-Insulin-Dependent Diabetes Mellitus; NIDDM). Unlike Type 1 diabetes mellitus, Type 2 is associated with hyperinsulinism. Primarily, overall physiologic distortions prompted by poor control of metabolism in absence or insufficiency of insulin engender hyperglycemia and associated metabolic disorders [1,2]. Striking consequential effects of prolong hyperglycemia are changes in structure and function of macromolecules [3,4], auto-oxidation of glycated proteins, increased production of reactive oxygen species (ROS), decreased antioxidant defense, increased lipid peroxidation, and associated apoptosis or necrosis occasioned by membrane degeneration [4,5]. Notably, alterations/adjustments in most glycolytic, tricarboxylic acid cycle (TCA) enzymes activities are associated with diabetic states [5,6]. Activities of these enzymes (pyruvate kinase, pyruvate dehydrogenase, glycogen synthase, pyruvate carboxylase, fructose 1, 6-bisphosphate etc.) are regulated by insulin and have been observed to be phosphoenzymes. Activation of enzyme activity in response to insulin stimulus is prompted by cyclic adenosine monophosphate (cAMP) phosphodiesterase mediated pathway [6] or through secondary metabolic events connected to insulin action.

Glutathione S-transferases (GSTs) are multi-gene and multifunctional antioxidant enzymes that comprise several classes of GST isozymes. These enzymes by virtue of their activities act as subset of numerous cellular antioxidants defense systems against ROS species that are associated with many disease-causing electrophiles [2,7,8]. NADH-Methaemoglobin reductase (NADH-MR) (EC: 1.6.2.2) transfers electrons from NADH + H^+^ to cytochrome *b*_5_ via its flavin adenine dinucleotide (FAD) prosthetic group [9]. This erythrocyte enzyme maintains hemoglobin in its ferrous (Fe^2+^) state [10]. Na^+^/K^+^-ATPase, also called the sodium pump, is a soluble conserved trimeric pump (α-133 kDa; β-35 kDa; γ-10 kDa) involved in transmembrane cation regulation via ATP–dependent dual efflux/influx of sodium (Na^+^) and potassium (K^+^) ions in various cells [11,12]. The regulation of this pump activity is dependent on the phosphorylation of the α-subunit of Na^+^/K^+^-ATPase [11,13].

*Allium sativa* has been widely reported to exhibit therapeutic benefits to numerous pathologic states whose etiology is linked to oxidative stressors and electrophiles [14] such as diabetes mellitus [15-18], atherosclerosis [19,20], hyperlipidemia [20,21] thrombosis [22], hypertension [23]. Phytochemical and biochemical profile of *A. sativa* has been reported elsewhere [24]. The present study was based on the premise that hyperglycemia is one of the various indicators and promoters of distortional haemostasis associated with diabetes mellitus. Therefore, we sought to investigate level of alterations in erythrocyte GST, NADH-MR and Na^+^/K^+^-ATPase activities of hypoglycemic rats treated with ethanol/water (1:2 *v/v*) extract of *A. sativa* as agent of glycemic control.

## Materials and methods

### Collection of plant specimen

Fresh samples of *A. sativa* were obtained in July, 2012 from local market at Umoziri-Inyishi, Imo State, Nigeria. The plant specimen was identified and authenticated by Dr. F.N. Mbagwu at the Herbarium of the Department of Plant Science and Biotechnology, Imo State University, Owerri, Nigeria. A voucher specimen was deposited at the Herbarium for reference purposes.

### Preparation of extract

Fresh bulbs of *A. sativa* were washed under a continuous stream of distilled water for 15 min and air-dried at room temperature for 5 h. The bulbs were chopped and further dried for 5 h in an oven at 60°C and subsequently ground with a ceramic mortar and pestle. Twenty-five grams (25 g) of pulverized specimen was suspended in 250 mL of ethanol/water mixture (1:2 *v/v*) in stoppered flasks and allowed to stand at −4°C for 24 h. The suspensions were filtered with Whatman No. 24 filter papers. The filtrate was concentrated in a rotary evaporator at 50°C and dried in vacuum desiccator. The yield was calculated to be 3.4% (*w/w*). The extract was finally suspended in phosphate buffered saline (PBS) solution (extract vehicle), osmotically equivalent to 100 g/L PBS (90.0 g NaCI, 17.0 Na_2_HPO_4_.2H_2_O and 2.43 g NaH_2_PO_4_.2H_2_O), and used in all the studies with doses expressed in mg/kg of body weight of the animals.

### Experimental animals

Male rats *Rattus norvegicus* (8–10 weeks old) weighing 150–200 g were generous gift from Professor A.A. Uwakwe (Department of Biochemistry, University of Port Harcourt, Nigeria). The rats were maintained at room temperatures of 25 ± 5°C, 30–55% of relative humidity on a 12-h light/12-h dark cycle, with access to water and food *ad libitum* for 2 weeks acclimatization period. The handling of the animals was in accordance with the standard principles of laboratory animal care of the United States National Institutes of Health (NIH, 1978).

### Induction of hyperglycemia and study design

Hyperglycemia was induced by a single intra-peritoneal injection of 0.1 mol/L alloxan monohydrate in PBS solution (pH = 7.4) at a dosage of 140 mg/kg. The animals were considered hyperglycemic when their blood glucose concentrations exceeded 250 mg/dL 72 h after alloxan treatment, which was in conformity with our previous study [24]. The animals were deprived of food and water for additional 16 h before commencement of treatment (control and test experiments) as described elsewhere [24].

A total of twenty four (24) rats were divided into six (6) groups of four (*n* = 4) each as follows:

• Group C1; Control-Normal (C/N): Normal rats received only PBS (Vehicle; 1.0 mL/kg/16 h, i. p.) for 64 h.

• Group C2; Control-Hyperglycemic (C/H): Hyperglycemic rats received PBS (Vehicle; 1.0 mL/kg/16 h, i. p.) for 64 h.

• Group T1; H_[*A. sativa*] = 1.0 mg/kg_: Hyperglycemic rats received *A. sativa* (1.0 mg/kg/16 h, i. p.) for 64 h.

• Group T2; H_[*A. sativa*] = 2.0 mg/kg_: Hyperglycemic rats received *A. sativa* (2.0 mg/kg/16 h, i. p.) for 64 h.

• Group T3; H_[*A. sativa*] = 4.0 mg/kg_: Hyperglycemic rats received *A. sativa* (4.0 mg/kg/16 h, i. p.) for 64 h.

• Group T5; H_[Glibenclamide] = 5.0 mg/kg_: Hyperglycemic rats received glibenclamide (5.0 mg/kg/16 h, i. p.) for 64 h.

### Measurement of fasting blood sugar

After alloxan treatment, blood samples were drawn from apical region of the tails of the rats i.e., at experimental *t* = 0 h and by carotid artery puncture at experimental *t* = 76 h for measurement of fasting blood sugar (FBS). Determination of serum level of FBS was by glucose oxidase method according to the Randox® kit manufacturer’s procedure (Randox® Laboratories Ltd. Ardmore, United Kingdom). Glibenclamide, a standard anti-diabetic agent is a product of Aventis Pharma. Ltd. Goa, India.

### Collection of blood and preparation of erythrocyte haemolysate

At the end of treatment, the animals were fasted for 12 h [15] and subsequently sacrificed according to United States National Institutes of Health approved protocols (NIH, 1978). Blood volume of 4.0 mL was obtained by carotid artery puncture using hypodermic syringe. The erythrocytes were separated from plasma by bench centrifugation for 10 min. The harvested erythrocytes were washed by methods of Tsakiris *et al.,*[25] as described by Chikezie *et al.,*[26]. Within 2 h of collection of blood specimen, 1.0 mL of harvested erythrocyte was introduced into centrifuge test tubes containing 3.0 mL of buffer solution pH = 7.4: 250 mM tris (hydroxyl methyl) amino ethane–HCl (Tris–HCl)/140 mM NaCl/1.0 mM MgCl_2_/10 mM glucose). The erythrocytes suspension was further centrifuged at 1200 *g* for 10 min and repeated 3 times. According to Chikezie [27], to remove platelets and leucocytes, the pellet was re-suspended in 3.0 mL of phosphate-buffered saline (PBS) solution (pH = 7.4) and passed through a column (3.5 cm in a 30 mL syringe) of cellulose-microcrystalline cellulose (ratio *w/w* 1:1) [28]. The eluted fraction was passed twice through a new column of cellulose-microcrystalline cellulose (ratio 1:1 *w/w*) to obtain erythrocyte suspension sufficiently devoid of leucocytes and platelets. Finally, erythrocytes were re-suspended in 1.0 mL of this buffer and stored at 4°C. The washed erythrocytes were lysed by freezing/thawing as described by Galbraith and Watts, [29] and Kamber *et al.,*[30]. The erythrocyte haemolysate was used for the determination of erythrocyte glutathione S-transferase (GST) and NADH-Methaemoglobin reductase (NADH-MR) activity.

### Erythrocyte haemolysate haemoglobin concentration

The cyanomethaemoglobin reaction modified method of Baure, [31] as described by Chikezie *et al*., [26] was used for measurement of haemolysate haemoglobin concentration. A 0.05 mL portion of erythrocyte haemolysate was added to 4.95 mL of Drabkins reagent (100 mg NaCN and 300 mg K_4_Fe(CN)_6_ per liter). The mixture was left to stand for 10 min at 25 ± 5°C and absorbance read at λ_max_ = 540 nm against a blank. The absorbance was used to evaluate for haemolysate haemoglobin concentration by comparing the values with the standard.

### Erythrocyte glutathione S-transferase

GST activity was measured by the method of Habig, [32] as described by Pasupathi *et al.,*[3] with minor modifications according to Chikezie *et al.,*[26]. The reaction mixture contained 1.0 mL of 0.3 mM phosphate buffer (pH = 6.5), 0.1 mL of 30 mM 1-chloro-2, 4-dinitrobenzene (CDNB) and 1.7 mL of distilled water. After pre-incubating the reaction mixture at 37°C for 5 min, the reaction was started by the addition of 0.1 mL of erythrocyte haemolysate and 0.1 mL of glutathione (GSH) as substrate. The absorbance was followed for 5 min at λ_max_ = 340 nm. The enzyme activity was expressed as erythrocyte GST activity in international unit per gram haemoglobin (IU/gHb) using an extinction coefficient (∑) of 9.6 mM^−1^ cm^−1^ in reaction in which 1 mole of GSH is oxidized (Eq. 1).

### Erythrocyte NADH-Methaemoglobin reductase

NADH-MR activity was assayed according to the method of Board, [33]. A mixture of 0.2 mL Tris–HCl/EDTA buffer pH = 8.0, 0.2 mL NADH and 4.35 mL of distilled water was introduced into a test tube and incubated for 10 min at 30°C. The content was transferred into a cuvette and the reaction started by adding 0.2 mL of K_3_Fe(CN)_6_/0.05 mL erythrocyte haemolysate. The increase in absorbance of the medium was measured at λ_max_ = 340 nm per min for 10 min at 30°C against a blank solution. NADH-MR activity was expressed in international unit per gram haemoglobin (IU/gHb) using an extinction coefficient (∑) of 6.22 mM^−1^ cm^−1^ in reaction in which 1 mole of NADH + H^+^ is oxidized (Eq. 1).

### Calculation of GST and NADH-MR activities

(1) $$E_{A}=\frac{100}{\mathrm{Hb}}\times \frac{\mathrm{OD}/\min}{\Sigma }\times \frac{Vc}{V_{H}}$$

Where,

E_A_ = Enzyme activity in IU/gHb

[Hb] = Haemolysate haemoglobin concentration (g/dL)

0. D/min = Change per min in absorbance at 340 nm.

V_C_ = Cuvette volume (total assay volume) = 1.0 mL.

V_H_ = Volume of haemolysate in the reaction system (0.05 mL).

### Erythrocyte ghost membrane preparation

A simplified procedure of DeLuise and Flier, [34] as reported by Iwalokun and Iwalokun, [35] was used for erythrocyte ghost membrane preparation. Briefly, 10 mL of ice cold 5 mM Tris/0.1 mM Na_2_EDTA (pH = 7.6) were added to test tubes containing buffy coat free–packed erythrocytes of test and control rats to achieve osmotic lysis. The resulting membranes were centrifuged at 20,000 *g* for 20 min at 4°C. The membrane suspensions were washed 3 times in 0.017 M NaCl/5 mM Tris–HCl, pH = 7.6 and 3 times with 10 mM Tris–HCl (pH = 7.5). The haemoglobin-free membrane suspension was finally stored at −20°C in 10 mM Tris–HCl buffer (pH = 7.5).

### Erythrocyte Na^+^/K^+^-ATPase

The erythrocyte total ATPase activity was determined by incubating 50 μL of ghost membrane suspension (~200 μg of membrane protein) of test and control rats with 5 mM Tris-ATP, 25 mM KCl, 75 mM NaCl, 5 mM MgCl_2_, 0.1 mM EDTA, 25 mM Tris–HCl (pH = 7.5) in 500 μL for 90 min at 37°C in a shaking water bath. The reaction was stopped by adding tricarboxylic acid (TCA) to a final concentration of 5% (*w/v*). After centrifugation for 20 min at 1,500 *g*, an aliquot of the supernatant was used to measure total inorganic phosphate liberated according to Fiske and Subbarow, [36] reaction. This assay was repeated in the presence of 200 μM methyldigoxin, an inhibitor of Na^+^/K^+^-ATPase activity. Total ATPase activity was expressed as micromole of inorganic phosphate liberated per milligram membrane protein per hour (μM pi/mg protein/h). The activity of Na^+^/K^+^-ATPase was subsequently determined by subtracting total ATPase activity in the presence of digoxin from enzyme activity in the absence of the inhibitor drug.

### Ghost erythrocyte membrane protein

Membrane protein was measured according to the method of Lowry *et al.,*[37] after solubilizing aliquots of ghost membrane suspension with 0.2% sodium dodecyl sulfate (SDS). Bovine serum albumin (BSA) (50–300 μg), product of Sigma Chemical Company, Saint Louis, Missouri, USA, was used as standard. Absorbance was measured with Beckmann D700 spectrophotometer (Beckmann, USA) at λ_max_ = 720 nm.

### Statistical analyses

The data collected were analyzed by the analysis of variance procedure while treatment means were separated by the least significance difference (LSD) incorporated in the statistical analysis system (SAS) package of 9.1 version (2006). The correlation coefficients between the results were determined with Microsoft Office Excel, 2010 version.

## Results and discussion

Table 1 showed that serum FBS levels of C/N rats ranged between 72.93 ± 0.82–95.12 ± 0.92 mg/dL, whereas the experimental rats without glycemic control (C/H) was between 249.41 ± 1.03–256.11 ± 1.23 mg/dL. These values represented decrease in serum FBS levels by 22.19 mg/dL and 6.7 mg/dL in C/N and C/H rats respectively within the experimental time of 76 h. At experimental time *t* = 0 h and *t* = 76 h, serum FBS levels of C/H was significantly (*p* < 0.05) higher than C/N rats. A cursory look at Table 1 showed that hyperglycemic rats treated with ethanol/water (1:2 *v/v*) extract of *A. sativa* exhibited comparative reduced serum levels of FBS, which was in a dose dependent manner. Serum FBS levels of hyperglycemic rats at *t* = 0 h was within the range of 255.64 ± 1.09–267.94 ± 0.92 mg/dL.

**Table 1:** Serum FBS levels of hyperglycemic rats with/without glycemic control

** [FBS] mg/dL**		
**Group**	***t*** **= 0 h**	***t*** **= 76 h**
C/N	95.12 ± 0.92^a^	72.93 ± 0.82^a^
C/H	256.11 ± 1.23^b^	249.41 ± 1.03^b^
H_[*A. sativa*] = 1.0 mg/kg_	255.64 ± 1.09^b,c^	125.11 ± 0.91^c^
H_[*A. sativa*] = 2.0 mg/kg_	261.13 ± 2.00^b,c,d^	129.32 ± 1.50^c,d^
H_[*A. sativa*] = 4.0 mg/kg_	267.94 ± 0.92^c,d,e^	132.61 ± 0.81^d,e^
H_[Glibenclamide] = 5.0 mg/kg_	265.49 ± 49^c,d,e,f^	101.12 ± 0.80^f^

Means in the column with the same letters are not significantly different at *p* > 0.05 according to LSD.

However, these values represented marginal variations in serum FBS levels amongst the three categories of *A. sativa* treated hyperglycemic rats (Group T1, Group T2 and Group T3) within the experimental time: 0 h ≤ *t* ≤ 76 h. Specifically, at *t* = 76 h, serum FBS_[*A. sativa*] = 1.0 mg/kg_ = 125 ± 0.91 mg/dL; FBS_[*A. sativa*] = 2.0 mg/kg_ = 129.32 ± 1.50 mg/dL and FBS_[*A. sativa*] = 4.0 mg/kg_ = 132.61 ± 0.81 mg/dL.; *p* < 0.05 compared to C/N rats. Furthermore, *t* = 76 h, the three groups of *A. sativa* treated hyperglycemic rats exhibited: H_[*A. sativa*] = 1.0 mg/kg_ = 51.06%, H_[*A. sativa*] = 2.0 mg/kg_ = 50.48% and H_[*A. sativa*] = 4.0 mg/kg_ = 50.41% reduction in serum FBS levels compared to their corresponding FBS levels at *t* = 0 h. Similarly, compared to serum FBS levels at *t* = 0 h, H_[Glibenclamide] = 5.0 mg/kg_ rats showed reduced serum FBS level by 61.91% at *t* = 76 h, representing a ratio of 1: 1.4 decrease in serum FBS levels compared to C/N rats; *p* < 0.05. At the end of the experiment, serum FBS levels of H_[*A. sativa*] = 1.0 mg/kg_ was not significantly different (*p* > 0.05) from H_[*A. sativa*] = 2.0 mg/kg_ rats. Likewise, FBS levels of H_[*A. sativa*] = 2.0 mg/kg_ showed no significantly difference (*p* > 0.05) compared to H_[*A. sativa*] = 4.0 mg/kg_ rats.

**Figure 1:**
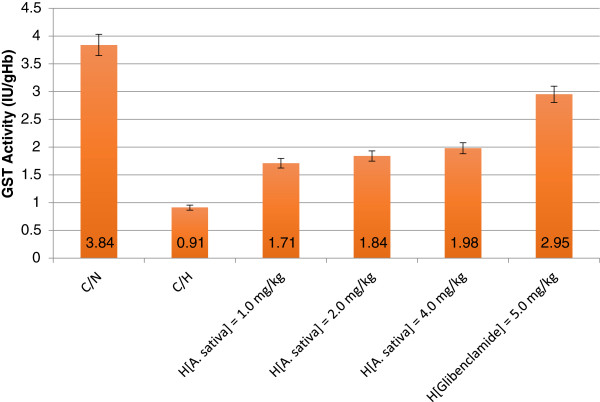
Erythrocyte GST activity of hyperglycemic rats with/without glycemic control.

Within the experimental time, hyperglycemic rats with or without glycemic control exhibited decreased levels of erythrocyte GST activity. Specifically, erythrocyte GST activity of C/H rats represented 23.69% of GST activity of C/N rats (*p* < 0.05). Figure 1 showed a corresponding increase in erythrocyte GST activity of hyperglycemic rats treated with ethanol/water (1:2 *v/v*) extract of *A. sativa* in a dose dependent manner (H_[*A. sativa*] = 1.0–4.0 mg/kg_) with comparative no significant difference (*p* > 0.05). Furthermore, within the experimental time, the decreased levels of erythrocyte GST activity of rats treated with ethanol/water (1:2 *v/v*) extract of *A. sativa* were significantly different (*p* < 0.05) from that of C/N rats. Also, erythrocyte of H_[Glibenclamide] = 5.0 mg/kg_ rats showed 76.82% GST activity compared to the C/N rats (*p* < 0.05).

**Figure 2:**
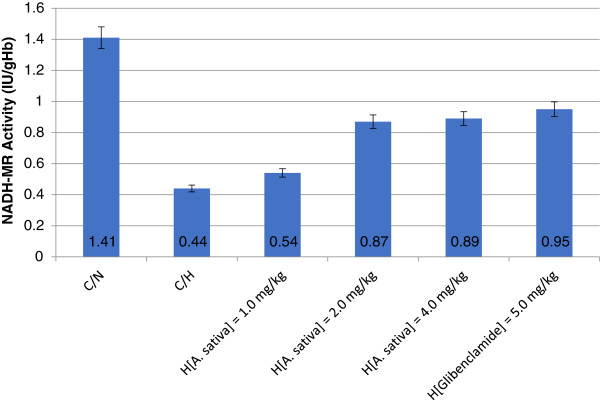
Erythrocyte NADH-MR activity of hyperglycemic rats with/without glycemic control.

Figure 2 showed that erythrocyte NADH-MR activity of rats without glycemic control (C/H rats) was not significantly different (*p* > 0.05) from those with glycemic control (H_[*A. sativa*] = 1.0–4.0 mg/kg_ and H_[Glibenclamide] = 5.0 mg/kg_ rats). Similarly, erythrocyte NADH-MR activity of H_[*A. sativa*] = 1.0–4.0 mg/kg_ rats was not significantly different (*p* > 0.05) from H_[Glibenclamide] = 5.0 mg/kg_ rats. Specifically, erythrocyte NADH-MR activity of H_[Glibenclamide] = 5.0 mg/kg_ rats was 67.38% compared to erythrocyte NADH-MR activity of C/N rats, whereas H_[*A. sativa*] = 1.0–4.0 mg/kg_ rats NADH-MR activity showed relative enzyme activity between the range of 49.65–63.12%. Erythrocyte NADH-MR activity of C/H rats was significantly (*p* < 0.05) lower than C/N rats, representing 68.97% reduction of NADH-MR activity in C/H rats.

**Figure 3:**
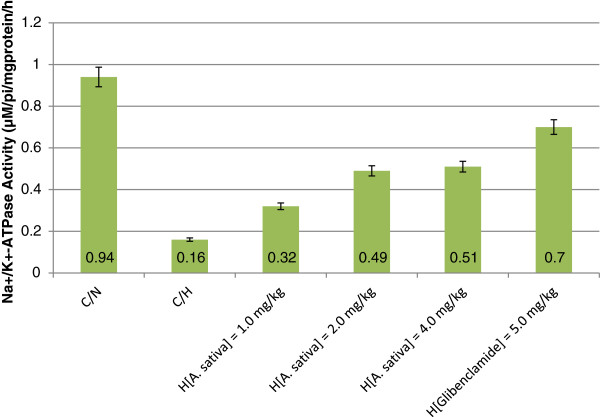
**Erythrocyte Na**^**+**^**/K**^**+**^**-ATPase activity of hyperglycemic rats with/without glycemic control.**

Erythrocyte Na^+^/K^+^-ATPase activity of C/H rats was reduced by 82.98% compared to C/N rats (*p* < 0.05). At experimental *t* = 76 h, erythrocyte Na^+^/K^+^-ATPase activity H_[*A. sativa*] = 1.0–4.0 mg/kg_ rats showed progressive increase in relative enzyme activity: H_[*A. sativa*] = 1.0 mg/kg_ = 34.04%, H_[*A. sativa*] = 2.0 mg/kg_ = 52.13% and H_[*A. sativa*] = 4.0 mg/kg_ = 54.26%. H_[Glibenclamide] = 5.0 mg/kg_ rats was 74.47% (Figure 3). An overview of Figures 1, 2 and 3 showed that the average relative activities of the three enzymes and corresponding order of enzyme activity in hyperglycemic rats treated with ethanol/water (1:2 *v/v*) extract of *A. sativa* was: NADH-MR = 60.99% > GST = 47.81% > Na^+^/K^+^-ATPase = 46.81%. In the same order, relative activities of the three enzymes in rats without glycemic control were: NADH-MR = 49.65% > GST = 23.69% > Na^+^/K^+^-ATPase = 17.02%. Furthermore, percentage decreases in GST, NADH-MR and Na^+^/K^+^-ATPase activities in H_[*A. sativa*] = 1.0–4.0 mg/kg_ rats was related to the capacity of ethanol/water (1:2 *v/v*) extract of *A. sativa* to exert dose dependent glycemic control.

The use of experimental animal model for study of Type 1 diabetes mellitus has been widely reported [38-41]. The cytotoxic action of diabetogenic agents is mediated by formation of superoxide radicals and other related ROS, causing massive destruction of the β-cells [39,42,43]. From the present study, experimental rats treated with the widely used diabetogenic agent–alloxan, in conformity with previous reports elsewhere [24,39,42,43], showed evidence of hyperglycemia (Table 1). Hyperglycemia is the earliest and primary clinical presentation in diabetic states [3,44]. Studies on the application of nutraceuticals, sourced from spices and other edible plants and their products, for the treatment and management of diabetes mellitus have received the attention of several research endeavours [45]. The present study showed evidence of the capacity of ethanol/water extract of *A. sativa* to reduce serum level of FBS in hyperglycemic rats, which compared fairly with the standard anti-diabetic drug-glibenclamide (Table 1). The anti-diabetic properties of *A. sativa* extract have been previously reported [15,24]. The therapeutic action of *A. sativa* as it applies to its role in the treatment and management of diabetes mellitus is identical to the mode of action of other numerous anti-diabetic agents of plant origin such as *Coriandrum sativum*[45]; *Gongronema latifolium*[46]; *Allium cepa* Linn [15]. However, other mechanism of therapeutic action, which involves increase peripheral glucose consumption induced by *Eugenia Floccosa*[47]*, Berberis lyceum*[48] and *Tinospora cordifolia* roots [49] have been documented. The active principles of these plant extracts exhibited insulin-like effect by mimicry. However, within the experimental time, administration of the three experimental doses of ethanol/water (1:2 *v/v*) extract of *A. sativa* as an instrument of glycemic control did not restore normal serum level of FBS (72.93 ± 0.82–95.12 ± 0.92 mg/dL) in hyperglycemic rats with [FBS] > 250 mg/dL.

According to Raza *et al.,*[5] oxidative stress is an important factor in the etiology and pathogenesis of diabetes mellitus. Furthermore, Pasupathi *et al.,*[3] had observed significant (*p* < 0.001) decrease in reduced glutathione (GSH) concentration in diabetic erythrocytes compared to control participants. They further averred that decreased level of GSH was an aftereffect of increased utilization of the coenzyme for scavenging ROS due to elevated oxidative stress associated with diabetes. Consequently, we observed decreased levels of erythrocyte GST activity in hyperglycemic rats, which was in conformity with previous studies [3,44,45,50-52], since the co-substrate (GSH) required for GST antioxidant protective activity [1,53] may have been utilized for other non-enzymatic reductive pathways. Judging from erythrocyte GST activity of rats without glycemic control, the relatively higher levels of erythrocyte GST activity of hyperglycemic rats treated with *A. sativa* extract in dose dependent pattern (Figure 1) was an obvious indication of the capability of ethanolic extract of plant extract to serve as anti-diabetic agent, fairly comparable to the standard anti-diabetic drug-glibenclamide. Erythrocyte GST activity has been proven to be a reliable biochemical index and basis for diagnosis and monitoring of therapeutic events in the course of treatment and management of other pathologic/metabolic disorders whose etiologies and manifestations are linked to oxidative stress. Notable among which are: parasitic infections [26,54], gout and rheumatoid arthritis [55,56], haemoglobinopathies [26], malignancy [57], hypertension [58], stroke [59] and atherosclerosis [60]. In a related perspective, Moasser *et al.,*[2] had previously given account of the use of GST activity as a reliable biomarker in depicting the etiology of diabetes mellitus. They posited that two isoforms of GST (GSTM1 and GSTT1) might be involved in the pathogenesis of Type 2 diabetes mellitus in South Iranian population. In addition, investigations by Yalin *et al.,*[7] showed that the GSTM1 gene may play a significant role in the aetiopathogeneses of diabetes mellitus and could serve as a useful biomarker in the prediction of diabetes mellitus susceptibility of the Turkish population.

According to Coleman, [61] poor glycemic control in diabetes and combination of oxidative, metabolic, and carbonyl stresses caused restriction in supply but excessive demand for reducing equivalents. Therefore, repressed NADH-MR activity in hyperglycemic rats could be linked to the substantial diversion and utilization of reducing equivalents to other reductive pathways in efforts to minimize oxidative stress, prompted by erythrocyte high ROS content. Thus, the decreased level of erythrocyte NADH-MR activity of hyperglycemic rats (Figure 2) is a reflection of a compromised erythrocyte antioxidant status associated with hyperglycemia [61,62]. Furthermore, in concordance with the present reports, Zerez *et al.,*[63] had stated that conditions that engender decreased erythrocyte NADH content resulted to decreased rate of methaemoglobin reduction in connection to impaired NADH-MR activity. This condition is responsible, in part, for relatively high methaemoglobin content in sickle erythrocytes and susceptibility to oxidative damage [27]. Based on the present observations, it is presumed that adjustments in diabetic erythrocyte methaemoglobin levels might provide early indication of diabetic antioxidant and oxidative stress status.

Studies suggest that insulin plays a stimulatory role in Na^+^/K^+^-ATPase activity through tyrosine phosphorylation process [11]. The relatively reduced levels of erythrocyte Na^+^/K^+^-ATPase activity in hyperglycemic rats (Figure 3) was consistent with the findings of previous authors. Soulis-Liparota *et al.,*[64] reported reduced Na^+^/K^+^-ATPase activity streptozotocin-induced diabetic rats with nephropathy, whereas, Di Leo *et al.,*[65] and Kowluru, [66] reported impairment in the enzyme activity in diabetic rats and mice with retinopathy. In a different study, using human participants, Iwalokun and Iwalokun, [35] noted compromised erythrocyte Na^+^/K^+^-ATPase activity in Type 1 diabetic patients from Lagos, Nigeria. This finding was corroborated by Mimura *et al.,*[67] study, in which they noted reduction of erythrocyte Na^+^/K^+^-ATPase activity in Type 2 diabetic patients with hyperkalemia. Raccah *et al.,*[68] suggested that diabetes-induced Na^+^/K^+^-ATPase activity dysfunction could be implicated in the pathogenesis of human diabetic neuropathy and the electrophysiological abnormalities.

The findings reported here was in concordance with those of Konukoglu *et al.,*[69]. They noted that hypercholesterolemia and free radical-induced mechanisms may be responsible for the inhibition of erythrocyte Na^+^/K^+^-ATPase activity in patients with Type 2 diabetes mellitus. According to the present study, decreased erythrocyte Na^+^/K^+^-ATPase activity of hyperglycemic rats was analogous to altered enzyme activity in peripheral neurons of individuals with diabetic neuropathy. According to Greene *et al.,*[70], impaired Na^+^/K^+^-ATPase activity is induced by hyperglycemia with characteristic distortions in myo-inositol and phosphoinositol metabolism, which normalizes with intensive insulin therapy that controls hyperglycemia [71]. Thus, decreased erythrocyte Na^+^/K^+^-ATPase activity was an obvious confirmation of a connection between the capacity of erythrocyte to actively transport Na^+^/K^+^ ions (antiport) and obligatory utilization of ATP for α-subunit of Na^+^/K^+^-ATPase phosphorylation required for enzyme activity [11,13,72]. Hyperglycemia with associated depressed glucose utilization in diabetic states results in low intracellular ATP concentration, insufficient for the required obligatory phosphorylation of the enzyme. The dose dependent increase in erythrocyte Na^+^/K^+^-ATPase activity in hyperglycemic rat treated with extract of *A. sativa* as instrument of glycemic control was an indication of improve glucose utilization exemplified in hyperglycemic rats treated with the standard anti-diabetic drug. The role and mechanism of insulin in regulation of Na^+^/K^+^-ATPase activity has been described elsewhere [73]. In another study, Konukoglu *et al.,*[69] reported that hypercholesterolemia and free radical-induced mechanisms may be responsible for the inhibition of erythrocyte Na^+^/K^+^-ATPase activity patients with type 2 diabetes mellitus.

The present study showed that erythrocyte GST, NADH-MR and Na^+^/K^+^-ATPase activities gave insights into the pathophysiology of diabetic state and could serve as a biomarker for ascertaining therapeutic control in Type 1 diabetes mellitus.

## Competing interests

The authors had no conflict of interest.

## Authors’ contributions

PCC wrote the draft and final manuscript of the reports, participated in the design and coordination of the study. AAU revised the manuscript, conceived and participated in the design and coordination of the study. All authors read and approved the final manuscript.
